# Virological Characteristics of Five SARS-CoV-2 Variants, Including Beta, Delta and Omicron BA.1, BA.2, BA.5

**DOI:** 10.3390/v15122394

**Published:** 2023-12-08

**Authors:** Yan Zeng, Fei Xia, Changfu Guo, Chunxia Hu, Yuwei Li, Xiang Wang, Qin Wu, Zhuo Chen, Jia Lu, Zejun Wang

**Affiliations:** Wuhan Institute of Biological Products Co., Ltd., Wuhan 430207, China; wyz140220@163.com (Y.Z.); 19907110481@163.com (F.X.); 13476229756@163.com (C.G.); huchunxiawh@126.com (C.H.); lwwei0407@126.com (Y.L.); wangxiang162723@163.com (X.W.); 18271682773@163.com (Q.W.); chenzhuo_zz@163.com (Z.C.)

**Keywords:** SARS-CoV-2, variants, Omciron BA.1, BA.2, BA.5, growth capacity, stability, infectivity

## Abstract

SARS-CoV-2 variants of concern (VOCs) show increasing transmissibility and infectivity and induce substantial injuries to human health and the ecology. Therefore, it is vital to understand the related features for controlling infection. In this study, SARS-CoV-2 WIV04 (prototype) and five VOCs (Beta, Delta, Omicron BA.1, BA.2 and BA.5 variants) were inoculated in Vero cells to observe their growth activities. Apart from evaluating the environmental stability at different temperatures, residual virus titers and infectivity at different temperatures (4 °C, room temperature (RT) and 37 °C) were measured over 7 days. The experiment also assessed the infectivity for different incubation durations. The growth capacity assay suggested that the WIV04, Beta and Delta variants replicated efficiently in Vero cells compared with Omicron Variants, and BA.2 replicated more efficiently in Vero cells than BA.1 and BA.5. In addition, all variants exhibited longer survivals at 4 °C and could remain infectious after 7 days, compared to RT’ survival after 5 days and at 37 °C after 1 day. The virus infection assay indicated that the Omicron variant had a weaker ability to infect cells compared to the WIV04, Beta and Delta strains, and a longer infection time was required for these strains, except for BA.2.

## 1. Introduction

Coronavirus Disease 2019 (COVID-19) is caused by Severe Acute Respiratory Syndrome Coronavirus 2 (SARS-CoV-2), an enveloped positive sense RNA virus ~30 kb of genomes in length [1,2]. Over 770 million confirmed cases and more than 6 million deaths cases have been reported in the word until 24 September 2023 (https://www.who.int/publications/m/item/covid-19-epidemiological-update---24-november-2023 (accessed on 25 November 2023)).

Notably, the rates of nucleotide substitution of RNA viruses are fast [3], which results in the accumulation of amino acid mutations, probably affecting viral transmissibility, pathogenicity and cellular tropism. This may pose great challenges in the development of diagnostic methods and valid vaccines. Although SARS-CoV-2 exhibits a decreased mutational rate compared with other RNA viruses, including Influenza and Human Immunodeficiency Virus (HIV), it has already been identified globally [4,5,6]. Since the first report of the D614G mutation [7], other mutations throughout the SARS-CoV-2 genome have been identified. B.1.351 represents the Beta variant initially discovered in South Africa [8]. B.1.617.2 (Delta) is the variant first obtained in India in December 2020 [9]. B.1.1.529 (Omicron) variant was first detected in November of 2021 in Botswana and South Africa and was designated as a VOC (Variant of Concern) on 26 November 2021 [10,11]. Then, Omciron BA.2, BA.3, BA.4, BA.5 and the more recently discovered XBB and EG.5 variants were reported [12,13,14,15].

The most notable gene mutations are those detected within the spike (S) proteins because of their ability to bind to angiotensin-converting enzyme 2 (ACE2) receptors in human host cells (Table 1). There are nine mutations in the S protein of the Beta variant (L18F, D80A, D215G, R246I, K417N, E484K, N501Y, D614G and A701V), including three mutations (K417N, E484K and N501Y) in the receptor-binding domain (RBD), with enhanced binding affinity for human ACE2 [16,17,18]. Mutations in the S protein of the Delta variant include T19R, L452R, T478K, D614G, P681R and D960N, and deletions at the 157 and 158 positions. Owing to the L452R mutation, the S protein exhibits an increased affinity for ACE2. The P681R mutation assists in cleaving the precursor S protein to activated forms referred to as S1 and S2 and contributes to enhanced virus–host cell fusion and integration [19,20]. At least 30 amino acid substitutions are detected in the S protein of the Omicron BA.1 variant, accompanied by three small deletions, and one tiny insertion (Ala67Val, Δ69-70, Thr95Ile, Gly142Asp, Δ143-145, Δ211, Leu212Ile, ins214EPE, Gly339Asp, Ser371Leu, Ser373Pro, Ser375Phe, Lys417Asn, Asn440Lys, Gly446Ser, Ser477Asn, Thr478Lys, Glu484Ala, Gln493Arg, Gly496Ser, Gln498Arg, Asn501Tyr, Tyr505His, Thr547Lys, Asp614Gly, His655Tyr, Asn679Lys, Pro681His, Asn764Lys, Asp796Tyr, Asn856Lys, Gln954His, Asn969Lys and Leu981Phe), among which 15 (residues 319–541) reside in the RBD. The above-mentioned important Omicron mutations could cause a higher transmission rate of the Omicron BA.1 variant because of the increased affinity for the ACE2 receptor [10]. BA.1 and BA.2 share many common mutations as well as the respective specific mutations. To be specific, BA.2 possesses eight unique mutations that cannot be detected in BA.1, while BA.1 has 13 specific mutations not found in BA.2 [21]. The BA.4 and BA.5 S proteins show the highest relation with BA.2. Apart from BA.2 mutations, mutations including 69–70del, L452R, F486V and wild type amino acid at position Q493 can be found in BA.4 and BA.5 [13,22].

While new variants are continuously emerging, some main variants have caused new waves of infections. The aggressive mutations of SARS-CoV-2 are related to virus replication rates, transmissibility, infectivity and eventually a severe disease phenotype. It is important to understand the virological characteristics of diverse variants to design and implement efficient and valid anti-SARS-CoV-2 strategies. Consequently, in this study, SARS-CoV-2 WIV04 (prototype) and five VOCs (Beta, Delta, Omicron BA.1, BA.2 and BA.5 variants) were inoculated in Vero cell to observe their growth activity. Apart from evaluating the environmental stability at different temperatures, residual virus titers and infectivity at different temperatures (4 °C, room temperature (RT) and 37 °C) were measured over 7 days. The experiment also assessed the infectivity of different incubation periods.

## 2. Materials and Methods

### 2.1. Cells Culture

Vero or Vero E6 cells were cultivated at 37 °C in a 5% CO_2_ atmosphere using Dulbecco’s modified Eagle medium (DMEM) (Gibco, Waltham, MA, USA) supplemented with 10% new-born bovine serum (NBS) (Sijiqing, Deqing, China).

### 2.2. Sources of SARS-CoV-2

The SARS-CoV-2 prototype strain used in this study was WIV04. hCoV-19/Wuhan/WIV04/2019(WIV04) is the official reference sequence employed by GISAID (EPI_ISL_402124) (https://gisaid.org/wiv04/ (accessed on 30 November 2023)). The SARS-CoV-2 variants used in this study included the Beta (B.1.351), Delta (B.1.617.2) and Omicron (B.1.1.529) BA.1, BA.2 and BA.5 variants. The WIV04 and all variants were obtained from Hubei, China, except for BA.1, which was obtained from Hong Kong, China. All viruses were cultured in the biosafety Level 3 laboratory of Wuhan Institute of Biological Products Co., Ltd. (Wuhan, China).

### 2.3. Virus Culture and Titration

Susceptible and permissive cells were used for producing viral stocks in vitro. Briefly, Vero cells were cultivated in a T-225 flask until they reached a 90–100% confluence. After discarding the medium, cells were washed twice with phosphate-buffered saline (PBS) (Sangon, Shanghai, China), followed by the addition of viral inoculum into the cell monolayer. Next, 40 mL of DMEM containing 2.5% NBS was added, and cells were incubated at 37 °C in a 5% CO_2_ atmosphere for 3 days. Finally, we obtained cell culture supernatant for 5 min centrifugation at 3000 rpm and preserved it under −80 °C prior to the analysis.

In titration assays, viruses were quantified in three replicates by titration using the same cells described above. Briefly, cells (2–3 × 10^4^/well) were inoculated into 96-well plates. The next day, serial 10-fold dilutions of viral inoculum were prepared in DMEM with 2.5% NBS and inoculated with a final volume of 100 µL per well. The plates were incubated at 37 °C in a 5% CO_2_ incubator for 3~5 days and cytopathic effects (CPEs) were visualized. Karber’s method was employed to calculate viral titers [23]. Viral titer (lgTCID_50_/0.1 mL) = L-d (s-0.5), where “L” is the log of the highest dilution and “d” represents the difference between the dilution log, while “s” stands for the sum of the positive tube ratio.

### 2.4. Virus Growth Capacity Assay

One day before infection, Vero cells were seeded into a six-well plate at a density of (1 × 10^6^) cells per well. Then, the cell culture medium was removed, the cells were washed twice with PBS and the viral inoculum was added to the cell monolayer at a 0.001 multiplicity of infection (MOI). The cells were incubated at 37 °C in a 5% CO_2_ atmosphere for 3 days. CPEs were visualized, nucleic acid copies were determined by Quantitative Real-time PCR (qRT-PCR) and viral titers were determined at 24~72 h post infection (h.p.i.).

### 2.5. Virus Stability Assay

The viruses were adjusted to a final concentration of 6.00 lgCCID_50_/mL by dilution in DMEM containing 2.5% NBS.

For the temperature stress assay, 10mL of the initial virus was incubated at different temperatures (4 °C, RT (21~22 °C) and 37 °C) for up to 7 days in three biological replicates. The samples were immediately titrated and inoculated into the cells to observe the infectivity of the remaining viruses at 0, 1, 3, 5 and 7 days.

For infectivity assays, Vero cells were seeded at a density of (1 × 10^6^ cells/well) in six-well plates one day prior to viral infection, using DMEM containing 10% NBS. After removing the supernatant on the following day, viral inoculum, which was incubated at different temperatures for 1, 3, 5 and 7 days, was inoculated in the cells of every well. The plates were incubated at 37 °C in a 5% CO_2_ atmosphere for 3 days, CPEs were visualized, and nucleic acid copies were determined by qRT-PCR.

### 2.6. Virus Infection Assay

On the day before the experiments, Vero E6 cells were seeded at a density of (4~5 × 10^5^ cells/well) in 12 multi-well plates. The viruses were diluted in DMEM containing 2.5% NBS at a MOI of 0.001 and then transferred into Vero E6 cells for 5, 15, 30 or 60 min at 3 °C with 5% CO_2_. At the end of the incubation, the viruses were removed, the cells were washed in PBS and 1 mL of new culture medium (containing 0.9% methylcellulose (Sigma, St. Louis, MO, USA) and 2% NBS) was added to the wells. The plates were incubated at 37 °C in a 5% CO_2_ atmosphere for 4 days. At the end of 4 days post-infection, supernatants were removed and cells were fixed in 4% paraformaldehyde (Sangon, Shanghai, China), and stained with 0.5% crystal violet (Sigma). After the staining procedure, image J software (image J 1.53k/Java 1.8.0 172 (64-bit)) was used to calculate the size of the plaque.

### 2.7. Quantitative Real-Time PCR

Commercially available kits were used following the specific protocols. The DA AN Nucleic Acid Extraction Kit (DA0623, DA AN Gene, Guangzhou, China) was employed for extracting viral RNA of 200 μL samples. qRT-PCR was performed using QuantStudio 5 real-time PCR system (Thermo Fisher Scientific, Waltham, MA, USA) by adopting SARS-CoV-2 virus nucleic acid assay kit (DA0992, DA AN gene, Guangzhou, China) which targets N gene of SARS-CoV-2.

### 2.8. Statistical Analysis

Data were plotted and analyzed using GraphPad Prism 8.0.2 software (GraphPad, Boston, MA, USA). A *t*-test was performed to compare two groups, whereas one-way analysis of variance was performed to compare several groups, with *p* < 0.05 indicating statistical significance. * *p*  <  0.05; ** *p*  <  0.01; *** *p*  <  0.001; **** *p*  <  0.0001; NS, no significance.

## 3. Results

### 3.1. Growth Capacity In Vitro

The growth capacity of SARS-CoV-2 prototype WIV04 and variants of the Beta, Delta and Omicron BA.1, BA.2 and BA.5 was evaluated by incubation in Vero cells.

**Figure 1:**
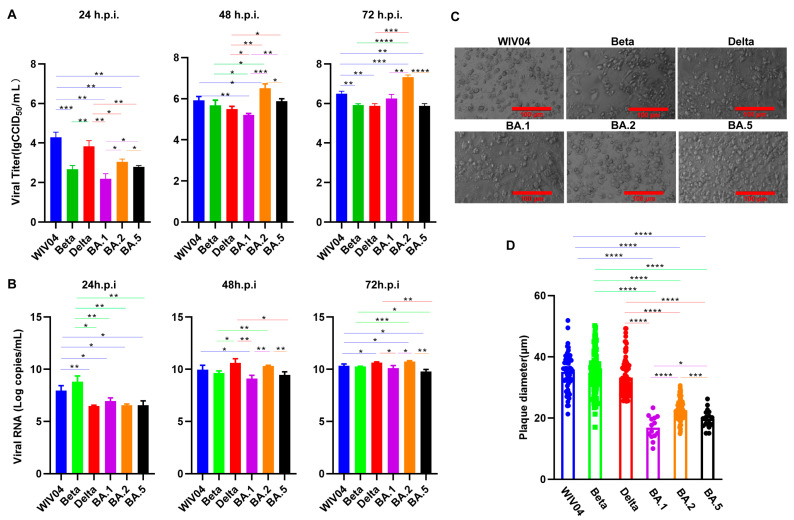
The growth capacity of the WIV04 and Beta, Delta and Omicron BA.1, BA.2 and BA.5 variants tested in Vero cells. (**A**) The viral titers at 24, 48 and 72 h.p.i. (**B**) The viral RNA copies at 24, 48 and 72 h.p.i. (**C**) CPEs of all variants within Vero cells at 72 h.p.i. (**D**) Plaques of all variants within Vero cells at 96 h.p.i. * *p*  <  0.05; ** *p*  <  0.01; *** *p*  <  0.001; **** *p*  <  0.0001.

For those tested variants, their replication kinetics were conducted in Vero cells (Figure 1A,B). From 24 to 72 h.p.i., viral titers and RNA level increased gradually. The viral titers of WIV04 and Delta increased rapidly to 48 h.p.i. and slowly from 48 to 72 h.p.i. The virus titers of Beta, BA.1, BA.2 and BA.5 increased slowly within 24 h.p.i., rapidly from 24 to 48 h.p.i. and slowly from 48 to 72 h.p.i. The nucleic acid copies of WIV04 increased rapidly to 48 h.p.i., and slowly from 48 to 72 h.p.i. The nucleic acid copies of Beta increased rapidly within 24 h and slowly from 24 to 72 h.p.i. BA.1, BA.2 and BA.5 increased slowly within 24 h.p.i., rapidly from 24 to 48 h.p.i. and slowly from 48 to 72 h.p.i. Titers of WIV04 and Delta variants significantly increased relative to Beta and Omicron variants at 24 h.p.i. At 48 h.p.i. and titers of WIV04, Beta, Delta and BA.5 variants were not significantly different; meanwhile, the variants were markedly higher relative to the BA.1 variant and lower compared with the BA.2 variant. At 72 h.p.i., titers of Beta and Delta variants were not significantly different, and were apparently lower than the BA.2 variant. Most interestingly, of those three Omicron variants, the titers of BA.2 were significantly higher than those of BA.1 and BA.5 from 24 to 72 h.p.i.

The CPE (Figure 1C) results showed that all variants caused CPEs in Vero cells. The CPEs caused by WIV04, Beta and Delta was more serious than those caused by BA.1, BA.2 and BA.5. In particular, the CPEs caused by BA.2 were more serious than by BA.1 and BA.5.

Plaques size between WIV04, Beta and Delta were not significantly different. Meanwhile, plaques generated after WIV04, Beta and Delta infections were larger than those formed by Omicron BA.1, BA.2 and BA.5 infections, while infections with BA.1 and BA.5 evidently decreased plaque size compared with BA.2 infection (Figure 1D).

The proliferation of WIV04, Beta and Delta Variants in Vero cells was not significantly different. Within 24 h.p.i., the proliferation efficiency significantly increased relative to Omicron variants. However, with the extension of the infection time, the titer of Omicron variants also increased gradually, and that of the BA.2 variant was evidently higher than that of other variants.

### 3.2. Stability at Different Temperatures

**Figure 2:**
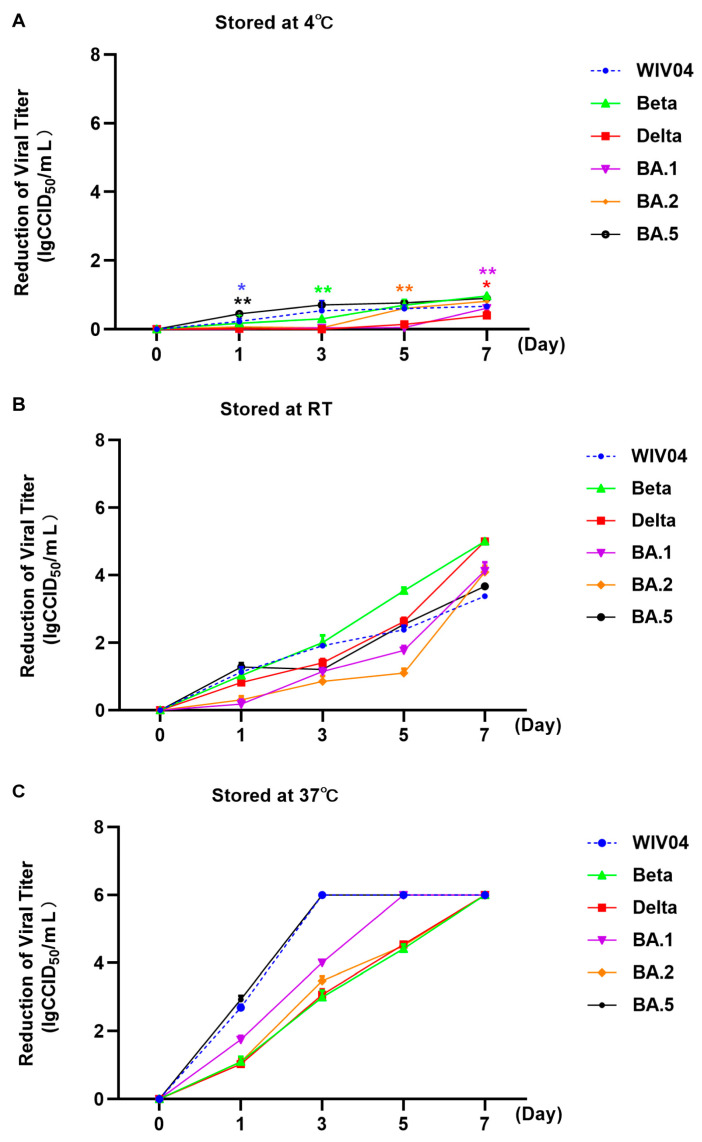
Stability of the virus titers of WIV04 and Beta, Delta and Omicron BA.1, BA.2 and BA.5 variants at different temperatures. (**A**) Reduction in viral titers after storage at 4 °C. (**B**) Reduction in viral titers after storage at room temperature. (**C**) Reduction in viral titers after storage at 37 °C. * *p*  <  0.05; ** *p*  <  0.01.

The stability of different variants of SARS-CoV-2 prototype WIV04 and variants of Beta, Delta and Omicron BA.1, BA.2 and BA.5 was evaluated by exposure to different temperatures, ranging from 4 °C to 37 °C (Figure 2).

After incubation with the initial viral titer of 6.00 lgCCID_50_/mL under 4 °C for 1, 3, 5 and 7 days, the viral titers decreased slightly as shown in Table 2 and Figure 2. According to statistical analysis, the titers of the WIV04 and the BA.5 significantly decreased from day 1, those of the Beta variants were significantly decreased from day 3, those of the Delta and BA.1 variants were significantly decreased from day 7 and those of BA.2 variant were significantly reduced from day 5.

At RT (Table 2 and Figure 2), all viral titers decreased significantly since day 1; in particular, the titers of the Beta and Delta variants on day 7 were below 1.00 lgCCID_50_/mL, so the reduction value was set as 5.00 lgCCID_50_/mL. In general, when stored at RT for 7 days, the WIV04 and Omicron variants were more stable than the Beta and Delta variants.

As for 37 °C (Table 2 and Figure 2), titers of all viruses decreased significantly from day 1. Markedly, most of the viral titers were out of the assay detectability, suggesting that viral particles almost lost infectivity and the reduction value was set as 6.00 lgCCID_50_/mL.

**Figure 3:**
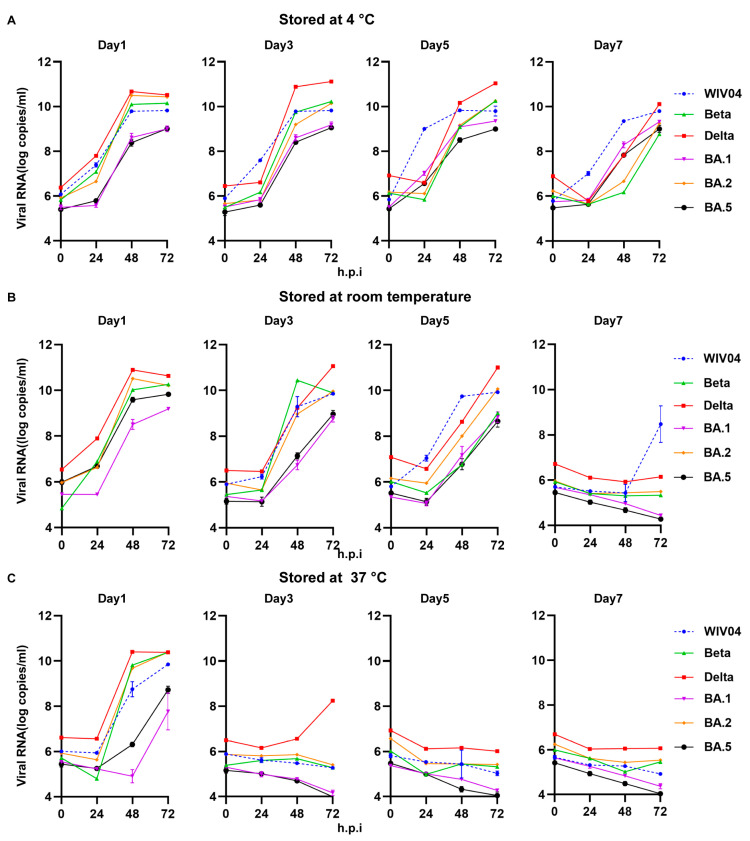
The infectivity of WIV04 and Beta, Delta and Omicron BA.1, BA.2 and BA.5 variants at different temperatures and different hours post infection (h.p.i.). (**A**) Replication kinetics of the remaining live viruses cultured in Vero cells for 24, 48 and 72 h.p.i. after storage at 4 °C for 1, 3, 5 and 7 days. (**B**) Replication kinetics of the remaining live viruses cultured in Vero cells for 24, 48 and 72 h.p.i. after storage at room temperature for 1, 3, 5 and 7 days. (**C**) Replication kinetics of remaining live viruses cultured in Vero cells for 24, 48 and 72 h.p.i. after storage at 37 °C for 1, 3, 5 and 7 days.

Infectivity under diverse temperatures 4~37 °C was also detected (Figure 3). After 7 days of storage under 4 °C, all viruses including WIV04, Beta, Delta and Omicron BA.1, BA.2 and BA.5 variants could still infect Vero cells and proliferate in them. At RT, the infectivity and replication activity of WIV04 and five variants were detected on days 1, 3 and 5. Significantly, these variants could hardly proliferate on day 7, except for WIV04. At 37 °C, the infectivity and replication activity were detected only on day 1 and the viruses could hardly proliferate on days 3, 5 and 7.

Combined with the experimental results, these data suggested that all variants exhibited longer survivals at 4 °C and showed stable infectivity by day 7, compared to the survival of RT by day 5 and 37 °C by day 1.

### 3.3. Infectivity In Vitro

**Figure 4:**
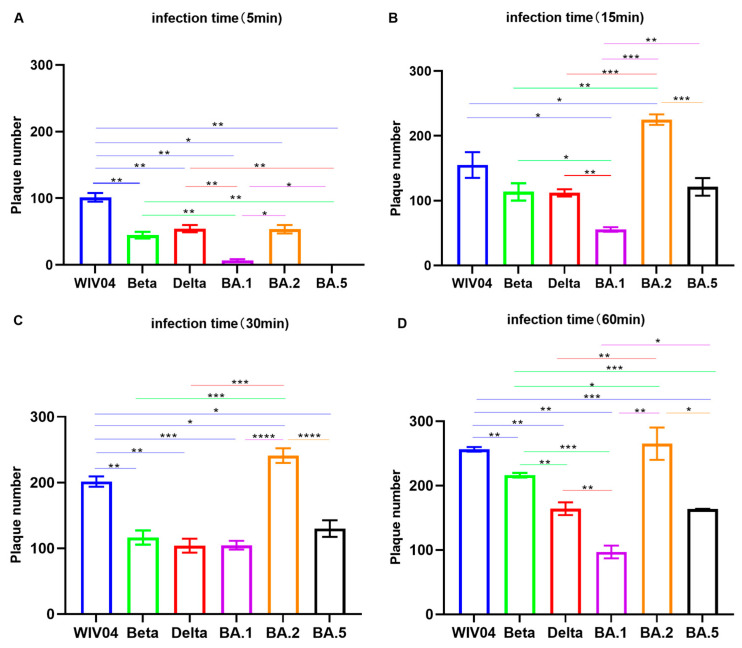
The infectivity of WIV04 and Beta, Delta, Omicron BA.1, BA.2 and BA.5 variants incubated in Vero E6 cells. (**A**) The number of plaques formed different virus strains were incubated in Vero E6 cells for 5 min. (**B**) The number of plaques formed different virus strains were incubated in Vero E6 cells for 15 min. (**C**) The number of plaques formed different virus strains were incubated in Vero E6 cells for 30 min. (**D**) The number of plaques formed different virus strains were incubated in Vero E6 cells for 60 min. * *p*  <  0.05; ** *p*  <  0.01; *** *p*  <  0.001; **** *p*  <  0.0001.

The infectivity of the SARS-CoV-2 variants Beta, Delta and Omicron BA.1, BA.2 and BA.5 were evaluated by incubation in Vero E6 cells over different periods of time, namely 5, 15, 30 or 60 min (Figure 4).

According to the findings, more plaques were formed by viruses when the incubation time was extended from 5 to 60 min. All strains could successfully infect cells to form plaques at 5 min, except for BA.5, which could not infect cells to form plaques at 5 min, and BA.1 only formed a few plaques. The ability of infected cells to form plaques of Beta, Delta and BA.1, BA.5 variants was weaker than that of the WIV04 in 5 min to 60 min. However, the ability of the BA.2 variant to form plaques in infected cells at 15 min was higher than that of BA.1 and BA.5and even higher than that of the WIV04, Beta and Delta variants.

These data indicated that the Omicron variant had a weaker ability to infect cells than that of the WIV04, Beta and Delta strains, and a longer infection time was required, except for BA.2 variant.

## 4. Discussion

The CPE induced by the Omicron strain was milder than those infected with the WIV04, Beta and Delta strains and the growth capacity experiments suggested that the Omicron variants grew slower than the above three virus strains within 24 h.p.i. The plaques size formed by the WIV04, Beta and Delta were larger compared with those formed by the Omicron variants. Our findings were consistent with the results reported by others [24]. Furthermore, our experiment showed that the BA.1 and BA.5 infections resulted in markedly decreased plaques compared with the BA.2 infections. Moreover, BA.2 was significantly more efficiently replicated than BA.1 and BA.5. BA.1 differed from BA.2 by 50 amino acids, which approximately doubles the number found among the four other variants of concern (Alpha, Beta, Gamma and Delta) and WIV04 [25]. Daichi Yamasoba et al. reported that the increased replicability and larger plaques formed by BA.2 compared with BA.1 and BA.2 exhibit an increased fusogenicity relative to BA.1 [25]. Compared with BA.2, BA.5 possesses three additional mutations (69–70del, L452R and F486V) and one reversion mutation (R493Q) within the S proteins. These are important for viral entry. Izumi Kimura et al. confirmed that rBA.5 demonstrated close growth to rBA.2 in Vero cells [26]. The difference in results might be that their studies used chimeric recombinant viruses, whereas our study used viruses isolated from naturally infected patients.

Other studies have reported that plaque formation size, replication dynamics and syncytia were correlated with fusogenicity [24,26]. Furthermore, in previous studies, authors reported that viral fusogenicity in in vitro cell cultures were closely related to the pathogenicity in in vivo hamster model [24,25,27]. Bo Meng et al. reported that Omicron compromised syncytia formation, which was consistent with reduced pathogenicity in vivo [28]. Scientists also discovered that pathogenicity in animal models is consistent with clinical data in humans [24,29,30,31,32,33,34]. Early assessment of the clinical severity of the SARS-CoV-2 revealed the risk of hospitalization or death was lower for Omicron cases [34,35]. Therefore, this present study may provide evidence that the Omicron variants showed a lower proliferation and pathogenicity than the WIV04, Beta and Delta variants tested, which is consistent with clinically mild symptoms in Omicron patients.

The environmental stability of the Wuhan strain was initially reported in 2020 [36,37,38]. According to some studies, Alpha and Beta variants exhibit an identical environmental stability level [39]. Moreover, the differences in stability between the Omicron and other strains were previously reported [40]. Researchers also suggested that analysis on individual temperature or concentration can be conducted to compare the environmental stability of different variants. Moreover, in real-life, viruses were released into the environment randomly. The differences in environmental stability at different temperatures between VOCs, like Beta, Omicron and Delta variants, remain unclear. Therefore, the stability of WIV04, Beta, Delta and Omicron BA.1, BA.2 and BA.5 variants was evaluated at 4 °C (Winter), RT (Spring and Autumn) and 37 °C (Summer) in this study. According to our findings, all variants were stable at 4 °C, when the temperature increased to RT or 37 °C, the viruses were quickly inactivated. Seasonal variations play a vital role in determining the time at which an infection might occur, its transmission and its potential to become an epidemic. Human coronaviruses are part of a group colloquially known as “winter viruses” which show peak incidences in the winter months. While it has been observed that seasonal changes have a significant impact on the transmissibility and infectivity of SARS-CoV-2, the exact mechanisms have not yet been elucidated until now. The result that all variants were stable at 4 °C may provide evidence for the higher transmissibility in “winter” and lower prevalence in “tropical and Southern temperate regions” [34,41,42,43]. Our study showed that the Omicron variant exhibited higher stability than the Beta and Delta variants at RT, and the stability decreased more slightly than the Beta and Delta variants by 1, 3, 5 and 7 days, consistent with the results of previous studies [40,44]. Omicron gained 100% prevalence in 3–4 months, much quicker than the six months it took for other VOCs to reach global or maximum prevalence levels [41]. Our study showed that Omicron variants have higher stability than the Beta and Delta variants at RT, probably facilitating them to replace the Delta variant and spread rapidly.

As announced by the WHO in May 2023, COVID-19 is not a public health emergency of international concern anymore. However, it remains controversial because novel subvariants continue to emerge and COVID-19 still poses a threat [45,46]. Since early August 2023, many countries have reported a novel Omicron subvariant called EG.5 and the WHO has upgraded it to a variant of interest [15]. Irrespective of the extent of disease severity, EG.5 remains concerning in terms of its growth advantage and immune evasion capability [47]. Similarly, in August 2023, BA.2.86, also a novel subvariant, was discovered and designated as a “variant under monitoring” [48]. The main difference between BA.2.86 and EG.5 is that the former has evolved from an earlier Omicron strain named BA.2 and has 33 S protein mutations and 14 RBD mutations compared with BA.2 [49,50]. The infectivity assay revealed that the Omicron variants required a longer infection time to infect Vero cells than the WIV04, Beta and Delta strains. This suggests that individuals can avoid infection by washing their hands frequently, wearing masks and other protective measures to reduce the time they spend in contact with the viruses. Currently, a low risk to public health caused by EG.5 and BA.2.86 is estimated, and SARS-CoV-2 is no longer an emergency, but it will be around for a long time. To prevent further EG.5 and BA.2.86 infection peaks, it may be of necessity to implement mask-wearing and frequent hands-washing.

## Figures and Tables

**Table 1:** Animo acid substitutions within the variant lineages.

WHO Label	Lineage	Spike Mutations of Interest
Beta	B.1.351	L18F, D80A, D215G, R246I, K417N, E484K, N501Y, D614G, and A701V
Delta	B.1.617.2	T19R, Δ157-158, L452R, T478K, D614G, P681R, D960N
Omicron BA.1	B.1.1.529	A67V, Δ69-70, T95I, G142D, Δ143-145, Δ211, L212I, ins214EPE, G339D, S371L, S373P, S375F, L417N, N440K, G446S, S477N, T478K, E484A, Q493R, G496S, Q498R, N501Y, Y505H, T547K, D614G, H655Y, N679K, P681H, N764K, D796Y, N856K, Q954H, N969K, L981F
Omicron BA.2	B.1.1.529	T19I, L24S, Δ25-27, G142D, V213G, G339D, S371F, S373P, S375F, T376A, D405N, R408S, L417N, N440K, S477N, T478K, E484A, Q493R, Q498R, N501Y, Y505H, D614G, H655Y, N679K, P681H, N764K, D796Y, Q954H, N969K
Omicron BA.5	B.1.1.529	Δ69-70, G142D, G339D, S373P, S375F, L417N, N440K, L452R, S477N, T478K, E484A, F486V, Q493, Q498R, N501Y, Y505H, D614G, H655Y, N679K, P681H, N764K, D796Y, Q954H, N969K

Note: “Δ” represents deletion mutation.

**Table 2:** Reduction in viral titers at different temperatures.

Stored Temperature	Stored Time (Days)	Mean Value of Reduction in Viral Titers (lgCCID_50_/_mL_)					
		WIV04	Beta	Delta	Omicron BA.1	Omicron BA.2	Omicron BA.5
4 °C	1	0.23	0.30	0.05	0.00	0.04	0.71
	3	0.54	0.17	0.08	0.04	0.01	0.90
	5	0.61	0.70	0.14	0.04	0.94	0.45
	7	0.67	0.97	0.40	0.62	0.82	0.77
RT	1	1.13	1.03	0.82	0.18	0.31	1.28
	3	1.92	2.01	1.40	1.15	0.85	1.21
	5	2.38	3.54	2.62	1.77	1.10	2.54
	7	3.38	5.00	5.00	4.13	4.08	3.67
37 °C	1	2.69	1.10	1.03	1.75	1.10	2.92
	3	/	3.00	3.07	4.01	3.47	/
	5	/	4.42	4.54	/	4.50	/
	7	/	/	/	/	/	/

Note: “/” represents the virus titer was below the assay detection.

## Data Availability

Data are contained within the article.
